# Ensemble of Deep Masked Language Models for Effective Named Entity Recognition in Health and Life Science Corpora

**DOI:** 10.3389/frma.2021.689803

**Published:** 2021-11-19

**Authors:** Nona Naderi, Julien Knafou, Jenny Copara, Patrick Ruch, Douglas Teodoro

**Affiliations:** ^1^ Information Science Department, University of Applied Sciences and Arts of Western Switzerland (HES-SO), Geneva, Switzerland; ^2^ Swiss Institute of Bioinformatics, Geneva, Switzerland; ^3^ Computer Science Department, University of Geneva, Geneva, Switzerland; ^4^ Department of Radiology and Medical Informatics, University of Geneva, Geneva, Switzerland

**Keywords:** named entity recognition, deep learning, patent text mining, transformers, clinical text mining, chemical patents, clinical NER, wet lab protocols

## Abstract

The health and life science domains are well known for their wealth of named entities found in large free text corpora, such as scientific literature and electronic health records. To unlock the value of such corpora, named entity recognition (NER) methods are proposed. Inspired by the success of transformer-based pretrained models for NER, we assess how individual and ensemble of deep masked language models perform across corpora of different health and life science domains—biology, chemistry, and medicine—available in different languages—English and French. Individual deep masked language models, pretrained on external corpora, are fined-tuned on task-specific domain and language corpora and ensembled using classical majority voting strategies. Experiments show statistically significant improvement of the ensemble models over an individual BERT-based baseline model, with an overall best performance of 77% macro F1-score. We further perform a detailed analysis of the ensemble results and show how their effectiveness changes according to entity properties, such as length, corpus frequency, and annotation consistency. The results suggest that the ensembles of deep masked language models are an effective strategy for tackling NER across corpora from the health and life science domains.

## 1 Introduction

In the health and life science domains, most of the information is encoded in unstructured reports. For example, it is estimated that around 90% of electronic health records (EHR) data are available as free text. While text format facilitates capturing information, it makes the secondary use of the data challenging. To support data structuring and to unlock the value of textual databases in secondary usage applications, named entity recognition (NER) methods have been proposed. NER is the task for detecting entities in text and assigning concept names, or categories, to them. The health and life science domains are notoriously known for their wealth of named entities and synonyms, such as microorganism taxonomies, drug brands, and gene names, to name a few. This richness of named entities together with the variety of formats, abbreviations, and (mis)spellings makes NER in health and life science corpora, like EHR, lab protocols, and scientific publications, a challenging task.

Basic NER approaches use the construction of dictionaries of named entities and the specification of tagging rules (Quimbaya et al., 2016). They normally require domain knowledge and feature engineering. While they are effective for simple and small corpora, their effectiveness is often limited when entities are complex and available in large numbers, as it is often the case in health and life sciences. Moreover, as the corpus evolves, it is hard to maintain the rules. More sophisticated methods are based on classical machine learning models, such as support vector machines, decision trees, hidden Markov models (Zhao, 2004), and conditional random fields (CRFs) (Li et al., 2008; Rocktäschel et al., 2012; Leaman et al., 2015). In these methods, annotated examples of text passages with entity classes are used to train the models. Textual features are combined with entity annotations to increase the model’s performance. As the models are trained only on the annotated corpus, which is usually small, they struggle to generalize to out-of-sample data. Thus, they are currently mainly used to provide a baseline for more effective model evaluation or in combination with more powerful models.

More recently, deep masked language models trained on large corpora have achieved state-of-the-art in most NLP-related tasks, including NER. Bidirectional Encoder Representations from Transformers (BERTs) (Devlin et al., 2019) were the first to explore the transformer architecture as a general framework for NLP (Vaswani et al., 2017). Once the model is trained (or pretrained in the BERTology parlance) on a large corpus, it can be adapted and effectively fine-tuned on specialized downstream NLP tasks, such as question-answering, text classification, and NER by leveraging the feature representations learned by the model during the pretraining phase in combination with examples of the specific task. Since the advent of BERT, a myriad of transformer-based masked language models have been proposed (Alsentzer et al., 2019; Liu et al., 2019; Yang et al., 2019). These models vary mostly in the tokenization used, in how the masking is performed, and in the trained data used during the pretraining phase. Language models pretrained on a specialized corpus, such as Medline, often tend to outperform models trained on a generic corpus for biomedical-related tasks.

In this study, our goal is to perform an empirical evaluation of how individual BERT-like models perform in the NER task across different health and life science corpora available in different languages. More specifically, we aim to assess how individual models compare to ensemble strategies in such scenarios. To do so, we leverage deep language models pretrained on the external text and fine-tune them on specific health and life science corpora. Then, their predictions are combined to create ensembles of named entity recognizers. We evaluate our models in chemistry, clinical, and wet lab corpora provided in the context of the ChEMU (Cheminformatics Elsevier Melbourne University) (He et al., 2020b), DEFT (Défi Fouille de Textes) (Grabar et al., 2018), and WNUT (Workshop on Noisy User-generated Text) (Tabassum et al., 2020) challenges, respectively. Our results show that the ensembles of named entity recognizers based on masked language models can outperform individual language models and achieve effective NER performances in these different domains and languages. We further perform an analysis of certain entity properties, including entity length, corpus frequency, and annotation consistency, to have a better understanding of the model’s performance.

## 2 Related Work

Deep learning approaches trained on large unstructured corpora have shown considerable success in NLP problems, including NER (Lample et al., 2016; Beltagy et al., 2019; Devlin et al., 2019; Jin et al., 2019; Liu et al., 2019; Andrioli de Souza et al., 2020). These models learn contextual token and sentence representations using often a self-supervised masked language algorithm, in which they attempt to predict masked tokens within sentences. This step is usually referred to as pretraining. The learned representations can then be reused in a supervised setting for downstream tasks, such as question-answering, NER, and text classification. For domain-specific tasks, models originally pretrained on general corpora, such as BERT, can be further pretrained or specialized on domain-specific corpora to improve the originally learned representations according to the domain specificity (Alsentzer et al., 2019; Lee et al., 2019; Gururangan et al., 2020). There exist also models pretrained only on domain-specific data (Beltagy et al., 2019; Gu et al., 2021), which reduces the overall training time as domain-specific corpora tend to be smaller in favor of lower generalization power. In both cases, in practice, those models are further trained or fine-tuned with task-specific examples. In this case, the model is no-longer trained to predict masked tokens but rather the actual NLP task, such as token classification in the case of NER.

Several models are proposed for cross-domain NER (Pan et al., 2013; Lin and Lu, 2018; Jia et al., 2019; Liu et al., 2020, 2021). These models are usually trained to leverage embeddings from the different domains via a transfer learning process to improve entity tagging. Only a few of these studies focus on health and life science NER. One study is that of Lee et al. (2018), in which the authors utilize the idea of transfer learning to identify named entities in the i2b2 2014/2016 corpus using a model trained on the MIMIC dataset. In this study, we adopt a different approach for the cross-domain problem. Instead of benefiting from joint named-entity learning, we investigate a methodology based on the ensemble of deep masked language models and show how it can be effectively applied across complex NER domains. Moreover, we believe this is the first work proposing a generic and robust approach for NER across chemical, clinical, and wet lab corpora available in English and French.

### 2.1 Chemical Named Entity Recognition

To further improve the performance of traditional approaches based on hand-crafted features for the extraction of chemical entities (Rocktäschel et al., 2012; Leaman et al., 2015; Habibi et al., 2016; Zhang et al., 2016; Akhondi et al., 2016), a number of studies leverage the power of word embeddings created using neural networks, such as word2vec (Mikolov et al., 2013), in combination with traditional approaches like CRF (Leaman et al., 2015; Rocktäschel et al., 2012) in a single recurrent network model, usually based on the long short-term memory (LSTM) architecture (Habibi et al., 2017; Corbett and Boyle, 2018; Zhai et al., 2019; Hemati and Mehler, 2019). These methods have shown a significant improvement over the traditional methods on multiple datasets, such as CHEMDNER patent (Krallinger et al., 2015a,b) and BioSemantics (Akhondi et al., 2014). For example, on the chemical domain, Habibi et al. (2017) report about 5% improvement in F1-score using an LSTM-CRF model with word embeddings over a CRF with BANNER features (Leaman and Gonzalez, 2008), such as part-of-speech and character n-grams. Zhai et al. (2019) extended the Bidirectional LSTM-CRF (BiLSTM-CRF) model with contextualized word representations of Embeddings from Language Models (ELMo) (Peters et al., 2018) and reported an F1-score improvement of 3.7 percentage point over BiLSTM-CRF and LSTM character models.

Recently, the ChEMU evaluation lab (He et al., 2020b) organized an information extraction task from patent documents for the identification of chemical compounds and their specific roles in chemical reactions. The named entities in this task consist of four categories, including *chemical compounds* involving in a chemical reaction, *conditions* of the chemical reaction, *yields* for the final chemical product, and *example labels*. Teams participating in the task were evaluated based on both strict and relaxed span matching conditions. Various approaches have been proposed in the competition, including rule-based models (Dönmez et al., 2020; Wang et al., 2020), BiLSTM-CNN-CRF (Dao and Nguyen, 2020; Mahendran et al., 2020), and transformer-based models (Copara et al., 2020b; Dönmez et al., 2020; Ruas et al., 2020).

### 2.2 Clinical Named Entity Recognition

Various NER challenges and shared tasks, such as the i2b2 and n2c2 NLP challenges (Uzuner et al., 2010; Suominen et al., 2013; Kelly et al., 2014; Bethard et al., 2015; Névéol et al., 2015; Henry et al., 2020), fostered the development of NER methods (De Bruijn et al., 2011; Jiang et al., 2011; Kim et al., 2015; Van Mulligen et al., 2016; El Boukkouri et al., 2019) for the clinical domain in different languages (Lopes et al., 2019; Sun and Yang, 2019; Andrioli de Souza et al., 2020; Schneider et al., 2020). The DEFT challenge proposed an information extraction task for the French clinical corpus, with entities distributed across four categories: *anatomy*, *clinical practices*, *treatments*, and *time* (Cardon et al., 2020). Several teams participated in the challenge and the proposed approaches relied on rule-based models (Lemaitre et al., 2020; Royan et al., 2020; Hiot et al., 2021), CRF-based models (Minard et al., 2020), and transformer-based models (Copara et al., 2020a; Nzali, 2020).

Similar to the chemical domain, word embeddings helped improve the recognition of entities in clinical corpora. Roberts (2016) used the combination of a general domain and in-domain word2vec embeddings and showed improvement over only in-domain embeddings. Using the i2b2 NLP dataset (Uzuner et al., 2011), El Boukkouri et al. (2019) showed that the concatenation of off-the-shelf ELMo contextualized representations (Peters et al., 2018) and word2vec embeddings trained on i2b2 task outperformed ELMo embeddings alone. Contextualized embeddings provided by ELMo were also used by Zhu et al. (2018). The authors used an ELMo version trained on medical articles from Wikipedia and clinical notes and reported the state-of-the-art on MIMIC-III. Wei et al. (2020) used three approaches to identify entities on n2c2 dataset: a CRF, a BiLSTM, and a joint BiLSTM-CRF model. They investigated different ensemble strategies to combine those models and found that the best results were achieved using a majority voting.

As in other NLP tasks, recent studies to extract entities from clinical corpora focus mostly on the use of deep masked language models. Si et al. (2019) trained BERT on MIMIC-III and showed further improvement over the previous models on MIMIC-III. Alsentzer et al. 2019 trained BERT and BioBERT (Lee et al., 2019), on MIMIC notes, and showed that Bio + Clinical BERT performed better than BERT and BioBERT trained on MedNLI dataset and i2b2 2010 datasets. Similarly, Schneider et al. (2020) demonstrated that the fine-tuned BERT using Portuguese clinical notes outperformed BERT trained on general corpora.

### 2.3 Wet Lab Named Entity Recognition

NLP approaches have only been applied to experimental protocols relatively recently (Soldatova et al., 2014; Kulkarni et al., 2018). Luan et al. (2019) introduced a model based on a dynamic span graph to jointly extract named entities and relations on wet lab protocols and other corpora. Wadden et al. (2019) built upon Luan et al. (2019)’s model by combining BERT and dynamic span graph. Dai et al. (2019) computed the similarity of the pretrained data and the data of the target application to investigate the effectiveness of pretrained word vectors. Their results showed that the word vector’s effectiveness depends on the vocabulary overlap of the source and target domains.

In contrast to the chemical and clinical domains, challenges and shared tasks are not as common for wet lab protocol corpora. Recently, WNUT-2020 (Tabassum et al., 2020) introduced a NER task for analyzing Wet Lab protocols. The task covers entity types from five categories of *Action*, *Constituents*, *Quantifiers*, *Specifiers*, and *Modifiers*. More than a hundred manually annotated protocols were used to evaluate the submissions of 13 teams. Most of the participants used NER models based on contextualized word representations (Knafou et al., 2020; Singh and Wadhawan, 2020; Sohrab et al., 2020; Vaidhya and Kaushal, 2020; Zeng et al., 2020). A few participants used CRF-based models (Acharya, 2020).

## 3 Material and Methods

### 3.1 Datasets

In this section, we present the datasets used to train and assess the individual and ensembles of masked languages models for the extraction of named entities in chemical, clinical, and wet lab domains. The first dataset, provided in the context of the ChEMU 2020 challenge, consists of a collection of English chemistry patents annotated with chemical reaction entities. The second dataset, provided in the context of the DEFT 2020 challenge, consists of a collection of French EHR notes annotated with clinical entities. Finally, the third dataset, provided in the context of the WNUT 2020 challenge, consists of English laboratory protocols annotated with wet lab entities.

#### 3.1.1 Benchmark for Chemical Entity Recognition—ChEMU 2020 Dataset

**FIGURE 1:**
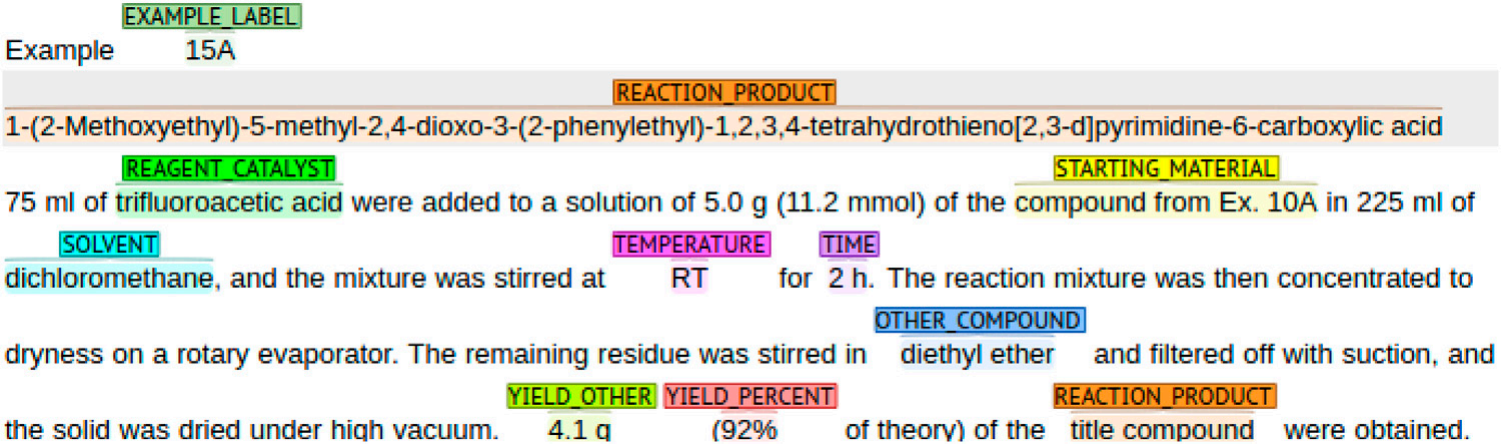
An example of a patent passage of the ChEMU dataset with entity annotations. The annotations are color-coded, representing the different entities in the dataset.

The ChEMU 2020 benchmark dataset contains snippets sampled from 170 English patents from the European Patent Office and the United States Patent and Trademark Office (He et al., 2020b,a, 2021; Verspoor et al., 2020). As shown in Figure 1, these snippets are annotated with several chemical reaction entities, including *reaction_product*, *starting_material*, and *temperature*. The training and test set of the ChEMU dataset contains a total of 1,500 snippets annotated with 26,857 entities using the BRAT standoff format (Stenetorp et al., 2012).

Table 1 shows the entity distribution for the training and test sets. The majority of the annotations are provided for the *other_compound*, *reaction_product*, and *starting_material* entities, covering 52% of the examples in the training and test datasets. In contrast, *example_label*, *yield_other*, and *yield_percent* entities represent together only 18% of entities in the training and test sets.

**TABLE 1:** Entity distribution in the official training and test sets of ChEMU benchmark dataset.

Entity		Train		Test	
		Count	%	Count	%
EL	example_label	1,104	5.5	349	5.2
OC	other_compound	5,720	28.3	1,931	28.9
RP	reaction_product	2,558	12.7	855	12.8
RC	reagent_catalyst	1,570	7.8	504	7.6
So	Solvent	1,390	6.9	428	6.4
SM	starting_material	2,167	10.7	711	10.7
Te	Temperature	1,861	9.2	612	9.2
Ti	Time	1,311	6.5	452	6.8
YO	yield_other	1,322	6.5	440	6.6
YP	yield_percent	1,183	5.9	389	5.8
	Total	20,186	100.0	6,671	100.0

#### 3.1.2 Benchmark for Clinical Entity Recognition—DEFT 2020 Dataset

**FIGURE 2:**
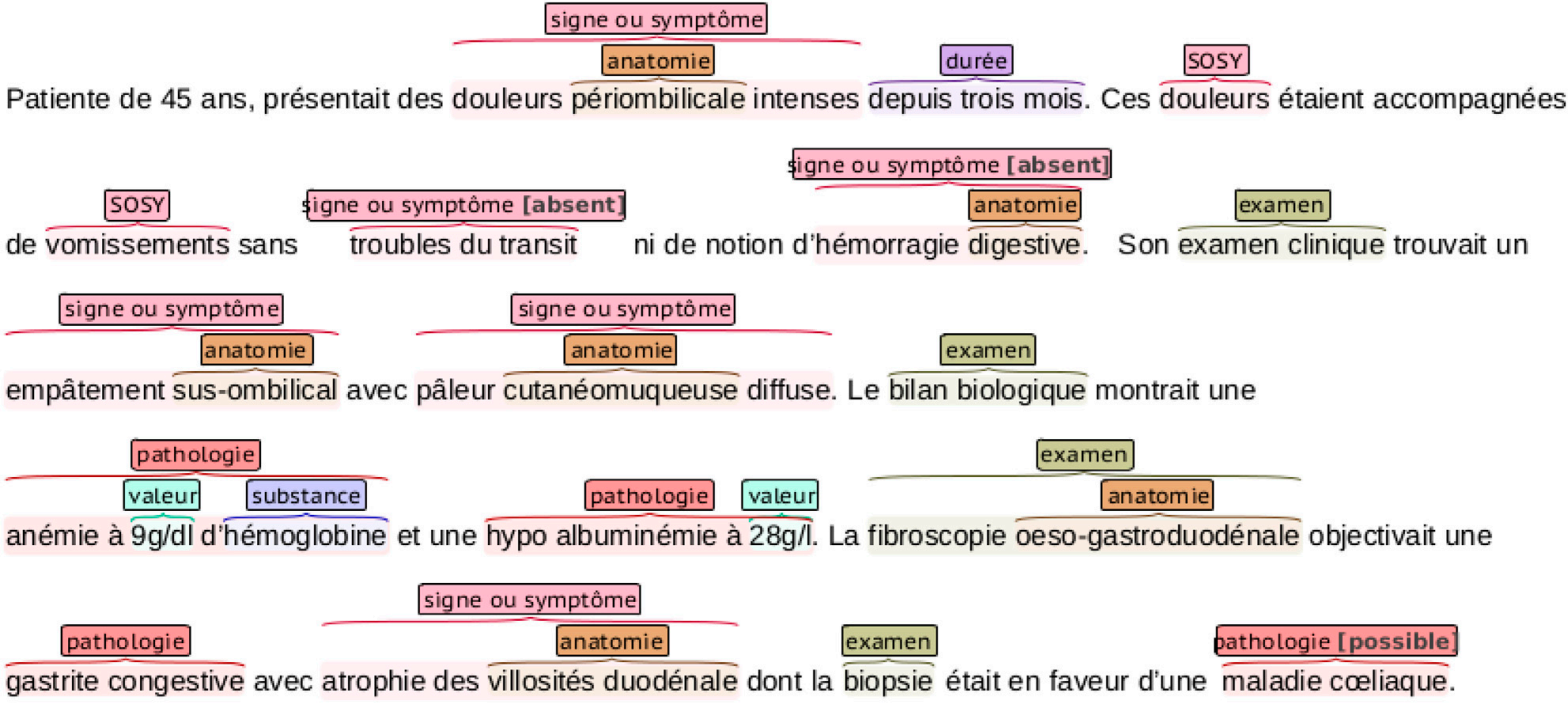
An example of a clinical narrative of the DEFT dataset with entity annotations. The annotations are color-coded, representing the different entities in the dataset. Notice that some entities are nested.

The DEFT benchmark dataset is a subset of the CAS corpus (Grabar et al., 2018), containing 100 French clinical documents manually annotated with the 8,098 entities in the following categories: *pathologie*, *sosy* (symptoms and signs), *anatomie*, *dose*, *examen*, *mode*, *moment*, *substance*, *traitement*, and *valeur*. An example of a clinical note annotation is shown in Figure 2. We can notice that nested entities appear in the annotations.

Table 2 shows the distribution of annotations among the entities in the training and test datasets. The majority of annotations come from the *sosy*, *anatomie*, and *examen* entities, which compose together 54% of the training data. On the other hand, *mode*, *dose*, and *pathologie* represent together only 13% of the training dataset. In contrast to the ChEMU data, the distribution of the training and test sets varies significantly.

**TABLE 2:** Entity distribution in the official training and test sets of the DEFT benchmark dataset.

Entity		Train		Test	
		Count	%	Count	%
**An**	Anatomie	1,298	17.5	174	25.7
**Do**	Dose	342	4.6	5	0.7
**Ex**	Examen	1,081	14.6	137	20.2
**Mod**	Mode	238	3.2	11	1.6
**Mom**	Moment	440	5.9	54	8.0
**Pa**	Pathologie	351	4.7	184	27.2
**So**	Sosy	1,647	22.2	33	4.9
**Su**	Substance	968	13.0	22	3.3
**Tr**	Traitement	494	6.7	52	7.7
**Va**	Valeur	562	7.6	5	0.7
	Total	7,421	100.0	677	100.0

#### 3.1.3 Benchmark for Wet Lab Entity Recognition—WNUT 2020 Dataset

**FIGURE 3:**
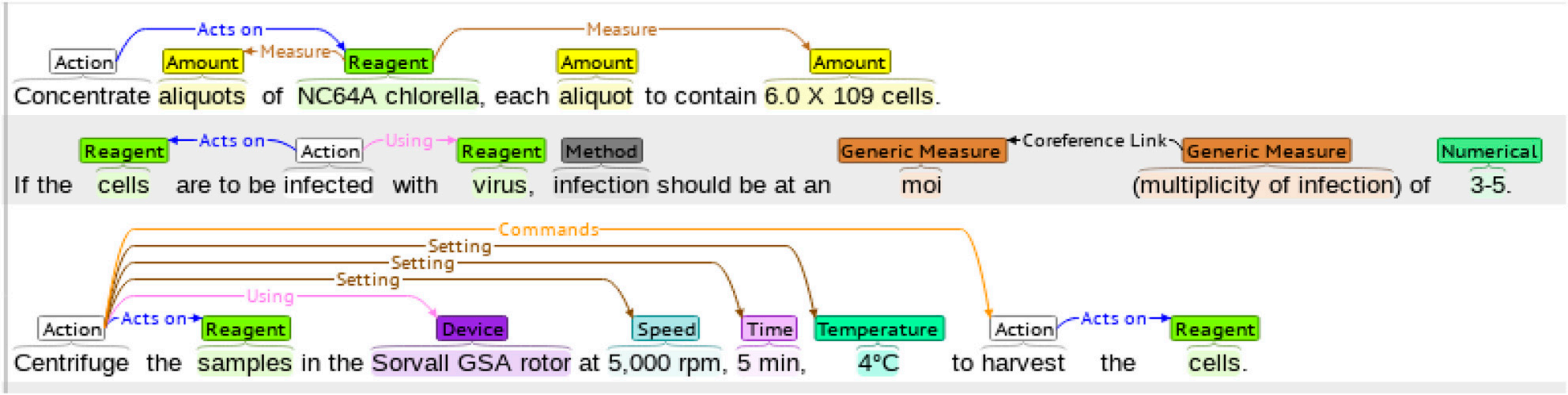
An example of a wet lab protocol of the WNUT dataset with entity annotations. The annotations are color-coded, representing the different entities in the dataset.

The WNUT benchmark dataset is composed of 727 unique English wet lab protocols that describe experimental procedures (Kulkarni et al., 2018). The dataset was manually annotated with the 102,957 entities in the following categories: *Action*, *Amount*, *Concentration*, *Device*, *Generic-Measure*, *Location*, *Measure-Type*, *Mention*, *Modifier*, *Numerical*, *Reagent*, *Seal*, *Size*, *Speed*, *Temperature*, *Time*, and *pH*. An example of a lab protocol annotation is shown in Figure 3.

In Table 3, we see the distribution of the 18 entities by each subset. As it is commonly found in the health and life science domains, there is a significant class imbalance, with only two classes (*Action* and *Reagent*) representing more than 50% of annotations in the training set. Similar to the ChEMU dataset, the proportions of entities are fairly similar across the training and test subsets.

**TABLE 3:** Entity distribution in the official training and test sets of the WNUT benchmark dataset.

Entity		Train		Test	
		Count	%	Count	%
Ac	Action	20,504	25.7	5,346	23.0
Am	Amount	5,712	7.2	1,223	5.3
Co.	Concentration	2,287	2.9	701	3.0
De	Device	2,836	3.6	888	3.8
GM	Generic-Measure	759	1.0	173	0.8
Lo	Location	6,643	8.3	1,657	7.1
MT	Measure-Type	1,453	1.8	720	3.1
Men	Mention	396	0.5	142	0.6
Met	Method	2,716	3.4	1,059	4.6
Mo	Modifier	7,736	9.7	3,416	14.7
Nu	Numerical	1,322	1.7	513	2.2
Re	Reagent	18,710	23.5	5,012	21.6
Se	Seal	366	0.5	119	0.5
Si	Size	498	0.6	232	1.0
Sp	Speed	1,032	1.3	238	1.0
Te	Temperature	2,610	3.3	744	3.2
Ti	Time	4,011	5.0	951	4.1
pH	pH	166	0.2	66	0.3
Total		79,757	100.0	23,200	100.0

### 3.2 Proposed Methodology

**FIGURE 4:**
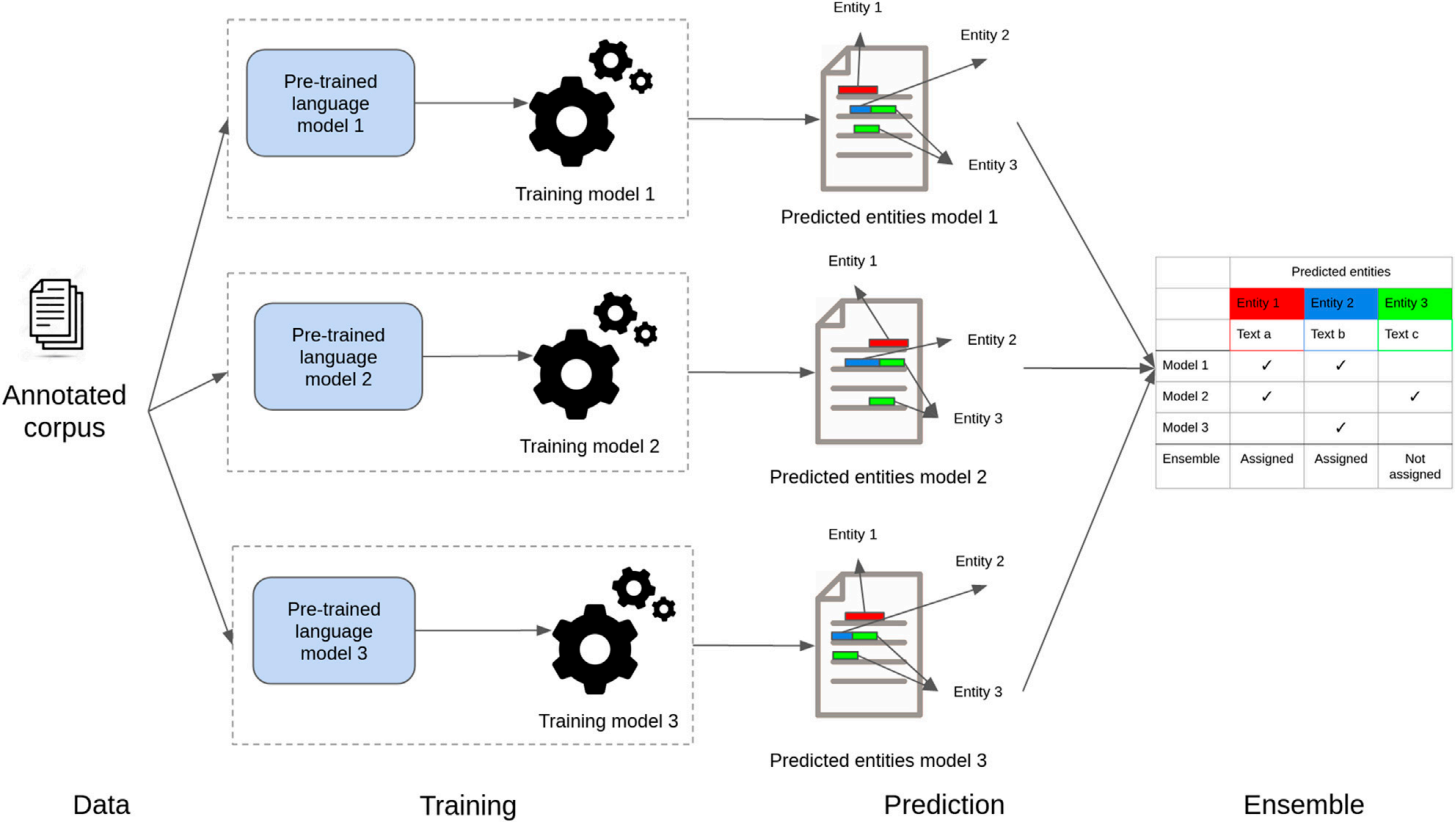
Schematic presentation of the ensemble model. Individual models are fine-tuned with specific task data. Then, they are used to classify tokens independently. The predictions are then combined using majority voting.

Figure 4 shows a high-level view of our proposed ensemble model to recognize entities in health and life science corpora. In step 1 (*data*), documents are preprocessed to create small text units using a sentence-splitting algorithm. In step 2 (*training*), the resulting sentences with entity annotations are used to fine-tune the individual deep neural masked language models. In the training process, sentences are tokenized according to the specific language model tokenizer algorithm, and each token is assigned a label (entity class label or no-entity) based on the training annotations. Then, in step 3 (*prediction*), sentences are fed to the individual models previously fine-tuned, which split them into tokens and assign an entity class. Finally, in step 4 (*ensemble*), the predictions created for each token are aligned using a majority voting algorithm.

In the following, we describe the methodology to fine-tune a single deep masked language model to recognize named entities in the chemical, clinical, and wet lab domains in English and French corpora. Then, we detail how these different fine-tuned language models were combined to provide an ensemble NER model.

#### 3.2.1 Single Deep Masked Language Model for Named Entity Recognition

To build the ensemble NER model, we fine-tuned different individual masked language models based on the transformers architecture (Vaswani et al., 2017). In the case of NER, masked language models are fine-tuned using a specialized training set—in our case, the chemical, clinical, and wet lab annotated corpora—to classify tokens according to the named entity classes. Table 4 lists the individual deep neural language models assessed in our experiments for each domain task. We used deep language models based on or derived from the BERT architecture. BERT was originally pretrained on a large corpus of English text extracted from BookCorpus (Zhu et al., 2015) and Wikipedia, with the different number of attention heads for the base and large types (12 and 24 transformer layers and hidden representations of 768 and 1,024 dimensions, respectively).

**TABLE 4:** Pretrained models used for NER in the ChEMU, DEFT, and WNUT benchmark datasets.

Dataset	Pretrained model	Model size	Corpus type
ChEMU	BERT-base-cased Devlin et al. (2019)	Base	general
	BERT-base-uncased Devlin et al. (2019)	Base	
	CNN	—	
DEFT	BERT-base-multilingual-cased	Base	—
	CamemBERT Martin et al. (2020)	Base	general
		large	
	CamemBERT-bio Copara et al. (2020a)	Base	bio + medical
		large	
WNUT	RoBERTa Liu et al. (2019)	Base	general
		large	
	XLNet Yang et al. (2019)	large	general
	BioBERT Lee et al. (2019)	—	bio
	Bio + Clinical BERT Alsentzer et al. (2019)	—	bio + clinical
	PubMedBERT Gu et al. (2021)	—	bio + medical
	BioMed RoBERTa Gururangan et al. (2020)	—	bio + medical

To fine-tune a particular masked language model for the NER task, we leverage the token representation created in its pretraining phase. A fully connected layer is added on top of the token representations and trained to classify whether a token belongs to a class or not. As transformers usually use tokenizers that work on word bits (or sub-tokens), during prediction, the entity label with the highest probability will be assigned to all sub-tokens of a word, and the sub-tokens will be then merged to build back the original word with the respectively assigned label. Finally, in a given sequence, if two adjacent words were given the same entity prediction, we would consider the two words as a phrase related to that entity.

Following this approach, the masked language model is then fine-tuned on the domain-specific data—chemical, clinical, and wet lab—using the training datasets previously discussed (ChEMU, DEFT, and WNUT). The fine-tuning is performed with the maximum sequence length of 265 tokens. The only preprocessing done was sentence-splitting. For the chemical and wet lab NER experiments, for which no nested entities were considered, we used a softmax function. Conversely, for the clinical NER, for which a token could be assigned to more than one entity, we used a sigmoid function to provide a multi-class classifier.

#### 3.2.2 Ensemble of Deep Masked Language Models for Named Entity Recognition

Our ensemble method is based on a voting strategy, where each model votes with its predictions and a simple majority of votes is necessary to assign the predictions (Copara et al., 2020b,a; Knafou et al., 2020). In other words, for a given document, our models infer their predictions independently for each entity (as shown in Figure 4). Then, a set of passages (token or phrases) that received at least a vote for the named entities is taken into consideration for casting votes. This means that, for a given document and a given entity, we end up with multiple passages associated with a number of votes. Then, again for a given entity, the ensemble method will assign labels to all the passages that get the majority of votes. Note that each entity is predicted independently and that the voting strategy allows a passage to be labeled as positive for multiple entities at once. Thus, our ensemble strategy is also capable of assigning labels to nested entities.

### 3.3 Experimental Setup

#### 3.3.1 Training Details

We conduct experiments using the three datasets listed in Tables 1, 2, and 3 for the individual models listed in Table 4. As shown in Table 5, we split the annotated collection into *train*, *dev*, and *test* sets and trained our models using subsets (*train* split) of the three datasets separately. The individual models of chemical, clinical, and wet lab NERs were fine-tuned on ChEMU, DEFT, WNUT train splits, respectively. The train, dev, and test sets were used to train the model weights, set the hyperparameters, and find the best ensemble configuration, respectively. The ensemble threshold for chemical and clinical NER was set to 3 and for wet lab NER to 4. More information about the fine-tuning of the models and the hyperparameter settings can be found in Copara et al. (2020b,a) and Knafou et al. (2020).

**TABLE 5:** Distribution of samples in the train, dev, and test collections for the different NER tasks. Train: collection used to train model parameters. Dev: collection used to tune model hyperparameters. Test: collection used to define the ensemble models. Blind test: collection used to evaluate models.

Dataset	Split	# Patent snippets	# EHR notes	# Wet lab protocols
Training	Train	800	80	370
Training	Dev	100	10	123
Training	Test	225	10	123
Test	Blind test	375	67	111

#### 3.3.2 Evaluation Details

A blind test set (*blind test* split in Table 5), provided as part of the official evaluation for the respective challenges, was used to evaluate our models. Results are reported using the competition official metrics—precision, recall, and F1-score—considering the exact span matching, that is, both the starting and the end offsets of the text spans of the predicted and gold standard reference entities must match. They were computed using the BRAT eval tool^1^, and the evaluation code was provided by WNUT organizers against the blind test set split. The ensemble models created for the different domains are compared to the respective individual language models participating in the ensemble. The Student’s t-test is used to assess the significance of the results. Results are considered statistically significant for *p*-values smaller than 0.05.

## 4 Results

### 4.1 Individual *vs*. Ensemble Models

Table 6 presents the NER results for the chemical, clinical, and web lab corpora obtained using the official blind evaluation set from the ChEMU, DEFT, and WNUT challenges, respectively. Individual model performance is compared with their respective ensembles for each corpus using the official challenge metrics. As the official test set of ChEMU is not yet publicly available, we also provide the performance of the ensemble and all its respective individual models on the official development set, taken as a blind test set, so that the chemical NER ensemble performance can be compared against all its individual models. In this case, the official training set was split into train and dev sets (as shown in Table 5). As we can notice, the ensemble models consistently outperform the individual models across the different domains and languages (English: ChEMU and WNUT; French: DEFT), with performance varying between 75.47 and 92.30% (considering only the official blind test evaluation). These results suggest that the ensemble strategy is a robust methodology for NER in the health and life science domains.

**TABLE 6:** Comparison of the ensemble model with the individual models on three datasets (ChEMU, DEFT, and WNUT). *Individual model was taken as reference for the individual model’s baseline. ** The official test set of ChEMU is not publicly available, so we report the results on the development set that was used as a test set (the training set was split into training and development sets). The comparison of individual models and ensemble for the DEFT and WNUT challenges are on the official test sets.

	Model	*P*	R	F1
**ChEMU**	Test set			
	BERT-base-cased*	90.83	**91.14**	90.98
	Ensemble (*t* = 3)	**93.78**	90.87	**92.30**
	Dev set**			
	BERT-base-cased	91.37	**91.44**	91.40
	BERT-base-uncased	90.93	91.33	91.13
	CNN	91.39	74.06	81.82
	Ensemble (*t* = 3)	**94.36**	91.39	**92.85**
**DEFT**	BERT-base-multilingual-cased*	68.62	69.27	68.94
	CamemBERT-base	71.93	69.72	70.81
	CamemBERT-large	74.12	**74.70**	74.41
	CamemBERT-bio-base	68.81	71.05	69.91
	CamemBERT-bio-large	73.74	73.67	73.70
	Ensemble (*t* = 3)	**78.75**	72.46	**75.47**
**WNUT**	BioBERT	78.45	72.66	75.44
	Bio + Clinical BERT*	77.09	71.44	74.16
	PubMedBERT	79.12	**73.70**	76.32
	RoBERTa base	76.66	70.69	73.55
	RoBERTa large	77.57	71.75	74.55
	BioMed RoBERTa	76.92	71.78	74.26
	XLNet	79.51	71.53	75.31
	Ensemble (*t* = 4)	**84.73**	72.25	**77.99**

Bold in shows the best results.

Considering each domain, the ensemble model on the chemical corpus outperforms its respective individual models, achieving 92.30% of the exact F1-score on the official blind test set and yielding 1.3 percentage point improvement over the BERT-base-cased baseline (*p* = 0.005). The ensemble model on the clinical corpus achieves an F1-score of 75.47%, outperforming the multilingual BERT baseline by 6.5 percentage point (*p* = 0.025). The best performance among the participating individual model in the clinical NER ensemble is achieved by CamemBERT-large, around 1.1 percentage point below the ensemble. The clinical NER has the worse performance among the different domains assessed. We believe it could be due to two factors. First, clinical corpora are notoriously complex, with many abbreviations and heterogeneous writing style, particularly compared to patents and protocols, in which documents are expected to follow a more formal structure and writing standard. Second, the clinical NER might suffer from the known problem of lack of resources for non-English languages (worsened in the case of clinical corpora). Similar to the other domains, the ensemble model on the wet lab corpus outperforms its respective individual models (*p* = 0.05), achieving an overall F1-score of 77.99%. Among the individual models, the best performance is achieved by the fine-tuned PubMedBERT followed by the fine-tuned BioBERT.

Interestingly, the best recall is achieved by individual models in all tasks assessed, though not consistently across individual models. We believe that by combining the individual models in the ensemble, we restrict the predictions, taking only the ones that are more likely true, having thus a significant positive impact on the precision with an eventual negative impact on the recall for some models. For the particular case of wet lab protocols, the best recall is achieved by the fine-tuned PubMedBERT. Among all the contextualized models, PubMedBERT is the only model trained on biomedical text from scratch, and consequently, it has a more specific vocabulary set (Gu et al., 2021), whereas the other models are first trained on the general text and then further pretrained on biomedical, medical, and clinical texts.

### 4.2 Comparison With State-of-the-Art

Table 7 shows the comparative results of our ensemble models against the teams participating in the ChEMU, DEFT, and WNUT competitions ranked by exact F1-score. The best results in the ChEMU competition were achieved by Wang et al. (2020), whose models were based on BioBERT fine-tuned on ChEMU data and BiLSTM-CRF. Their predictions were further post-processed using hand-written rules, a step missing in our pipeline. Dao and Nguyen (2020) used BiLSTM-CNN-CRF with Word2Vec and Elmo embeddings trained on patent data. Ruas et al. (2020) also used BioBERT fine-tuned on ChEMU data to extract the entities. BioBERT is trained on PubMed and PMC, and these datasets provide a better pretraining dataset for the chemical domain than the General book and Wikipedia datasets. The competition baseline model is presented as BANNER (He et al., 2020b). Our ensemble model presented as *Ours* outperforms the BANNER baseline by 3.37% in terms of exact F1-score.

**TABLE 7:** Test phase results of the ensemble model compared to other participants for datasets of ChEMU, DEFT, and WNUT challenges.

	Team	P	R	F1
**ChEMU**	Wang et al. (2020)	**95.71**	**95.70**	**95.70**
	Dao and Nguyen (2020)	94.62	94.05	94.33
	Ruas et al. (2020)	93.27	94.57	93.92
	Ours	93.78	90.87	92.30
	Lowe and Mayfield (2020)	90.42	89.24	89.83
	BANNER Baseline He et al. (2020b)	90.71	87.23	88.93
**DEFT**	Wajsbürt et al. (2020)	**79.50**	**73.30**	**76.30**
	Ours	78.80	72.50	75.50
	Minard et al. (2020)	83.90	61.30	70.80
	Royan et al. (2020)	69.50	57.30	62.80
	Cao et al. (2020)	41.50	31.40	35.80
**WNUT**	Ours	**84.73**	72.25	**77.99**
	Singh and Wadhawan (2020)	81.36	**74.12**	77.57
	Sohrab et al. (2020)	83.69	70.62	76.60
	Kabir	78.79	72.20	75.35
	Vaidhya and Kaushal (2020)	77.00	72.93	74.91
	BIO-BIO	78.49	71.06	74.59
	Zeng et al. (2020)	76.21	71.76	73.92
	SudeshnaTCS	74.99	71.43	73.16
	B-NLP	77.95	63.93	70.25
	Acharya (2020)	73.68	63.98	68.48
	IBS	74.26	62.55	67.90
	DSC-IITISM	64.20	57.07	60.42
	mahab	50.19	52.96	51.54

Bold in shows the best results.

On the clinical dataset, our ensemble model achieved the second place in terms of F1-score. The best performing model in this corpus relied on a BiLSTM-CRF model and features provided by contextualized embeddings of the CamemBERT model (Wajsbürt et al., 2020). Finally, our ensemble model on the wet lab dataset achieved the best performance among the participants in terms of F1-score. The next model was based on BiLSTM-CRF architecture and features provided by the contextualized word embeddings of PubMedBERT (Singh and Wadhawan, 2020). As we can notice, the addition of a BiLSTM-CRF layer also provides a consistently high-performing strategy in such domains.

### 4.3 Entity Type Performance

Table 8 shows the performance of our ensemble models for all classes in the chemical, clinical, and wet lab NER tasks. In the chemical NER, the performance of the ensemble model ranges between 87% for *starting_material* and 99.74% for *yield_percent*. Error analysis on the training data shows that the *starting_material* entity is often confused with the *reagent_catalyst* entity. From the chemistry point of view, both starting material (reactants) and catalysts (reagents) entities are present at the start of the reaction, with the difference that the latter is not altered by the reaction. These terms are often used interchangeably though, which could be the reason for the confusion.

**TABLE 8:** Performance of the ensemble models in terms of exact precision, recall, and F1-score for the entities of the ChEMU, DEFT, and WNUT official test sets.

ChEMU				DEFT				WNUT			
Entity	*P*	R	F1	Entity	*P*	R	F1	Entity	*P*	R	F1
EL	97.11	96.28	96.69	An	79.60	81.80	80.69	Ac	91.17	84.43	87.67
OC	91.97	86.59	89.20	Do	60.00	46.15	52.17	Am	79.52	93.13	85.79
RP	89.42	85.96	87.66	Ex	76.39	70.50	73.33	Co.	88.40	90.78	89.57
RC	92.68	87.90	90.22	Mod	81.36	53.93	64.86	De	82.20	57.30	67.53
So	96.20	94.63	95.41	Mom	85.71	72.73	78.69	GM	57.02	39.20	46.46
SM	88.86	85.23	87.01	Pa	57.50	55.42	56.44	Lo	70.89	68.98	69.92
Te	97.69	96.90	97.29	So	71.98	63.25	67.33	MT	80.70	50.34	62.01
Ti	98.46	99.12	98.79	Su	77.27	54.31	63.79	Men	70.51	75.86	73.09
YO	97.76	99.09	98.42	Tr	67.47	55.26	60.76	Met	65.71	38.07	48.21
YP	99.74	99.74	99.74	Va	87.26	84.03	85.61	Mo	84.28	42.88	56.84
—	—	—	—	—	—	—	—	Nu	64.78	39.62	49.16
—	—	—	—	—	—	—	—	Re	85.71	85.69	85.70
—	—	—	—	—	—	—	—	Se	81.58	78.15	79.83
—	—	—	—	—	—	—	—	Si	69.12	19.75	30.72
—	—	—	—	—	—	—	—	Sp	86.19	85.83	86.01
—	—	—	—	—	—	—	—	Te	98.12	89.47	93.60
—	—	—	—	—	—	—	—	Ti	94.62	89.89	92.19
—	—	—	—	—	—	—	—	pH	98.39	92.42	95.31

In the clinical NER, the highest F1-score in the blind test set is achieved for the *valeur* entity (85.61%). This entity represents 7.6% of the annotations in the training collection. One could assume that entities with annotation examples above this threshold would perform well; however, when looking at the results for the *substance* (13.0% of the annotations) category, we notice an important drop in performance (63.79%). Thus, it seems that the number of training data examples alone is not sufficient to learn an entity automatically. The lowest performance for the ensemble method is found for the *dose* entity. This can be due to the variety of values in the annotated data, combining numbers and words (e.g., *de 0,5 à 0,75 L*), measure units (e.g., *1 mg/kg/j*), or simply words that could be easily associated with a nonentity word (e.g., *24 paquets/année* or *02*).

The performance of the ensemble model for the classes of wet lab NER ranges between 30.72 and 95.31%. Surprisingly, the entity with the highest F1-score, *pH* (95.31%), has only 0.2% of the annotations in the training sample. Again, the number of examples is not associated with the performance of the test set. Indeed, the best performing entities for the wet lab NER—*Temperature*, *Time*, and *pH*—are responsible together for only 8.5% of the annotation examples. The performance of the ensemble model is low for the *Generic-Measure*, which is similar to *dose* in clinical NER task, getting various forms, such as measure units (*volume*), measurements (*30* *kDa, 2.5 bars,* ∼*250–500* *bp*), and ratios (*1:2, 1/500 to 1/1,000*), which could also justify its low score.

### 4.4 Entity Property Analyses

**FIGURE 5:**
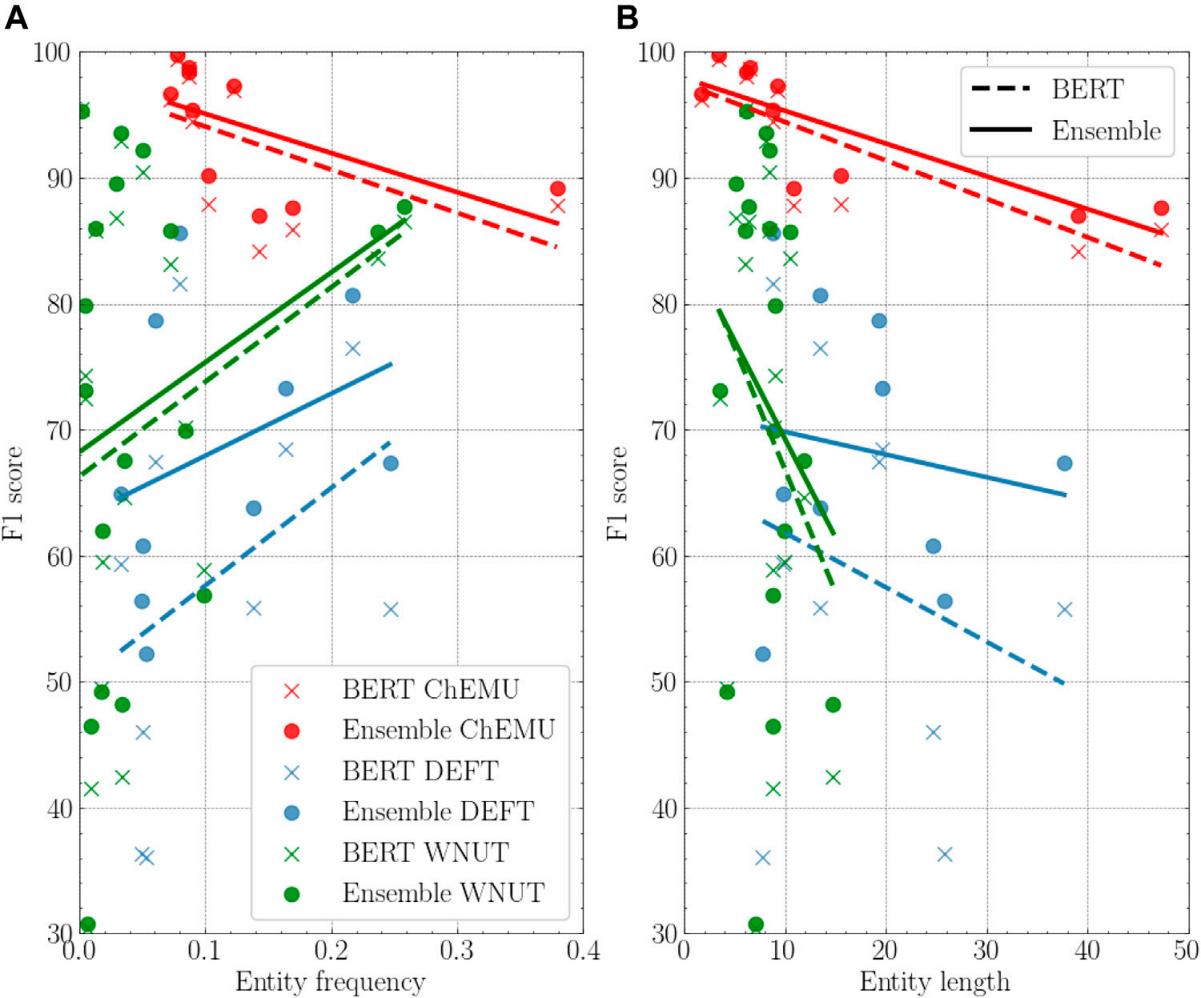
**(A)**: Performance of the BERT model *vs.* the ensemble model based on the entity frequency on the training data. **(B)**: Performance of the BERT model *vs.* the ensemble model based on the entity length on the training data. In both, the individual BERT for the three datasets is BERT-base-cased for ChEMU, BERT-base-multilingual-cased for DEFT, and Bio + Clinical BERT for WNUT.

To better understand our results across the different corpora, we performed a deeper analysis of the reference individual baseline and the ensemble model using different entity properties: frequency, length, and label consistency. Figure 5 shows the comparison of the BERT baselines and the ensemble models based on the entity frequency and length. For both DEFT and WNUT collections, on average, the highest performance gains over the individual model happen for the less frequent entities, whereas the opposite happens for the ChEMU collection. Concerning the entity length property, we notice that the average length is shorter in the WNUT dataset. The ChEMU dataset, as expected, includes the longest average entity lengths, necessary to represent molecules. For all datasets, as the entity length increases, the performance of the ensemble models improves over the individual models.

**FIGURE 6:**
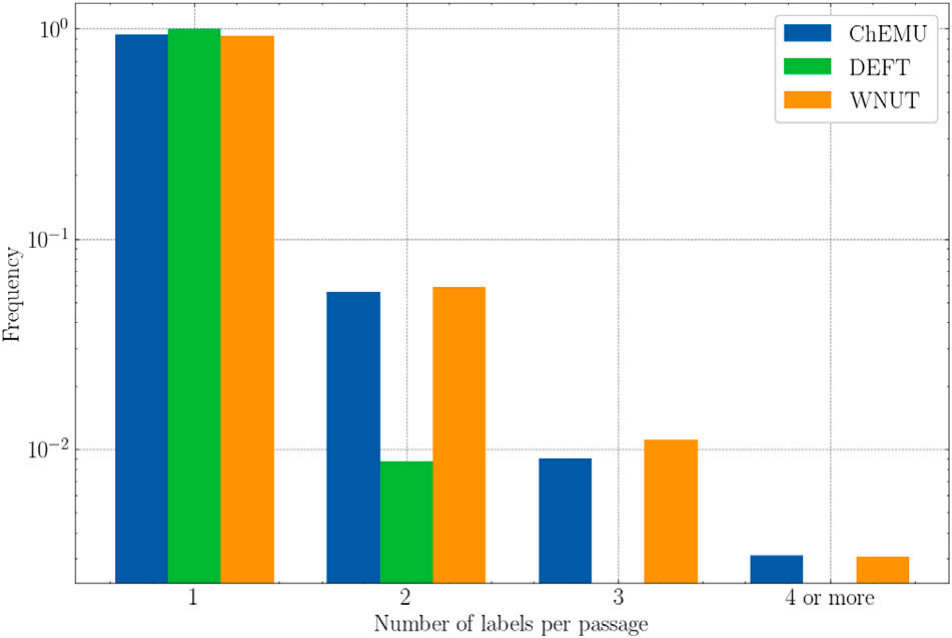
The number of labels assigned to each passage for the training set of the three datasets (ChEMU, DEFT, and WNUT).

Finally, Figure 6 shows the frequency of passages that were assigned more than one label for the evaluated datasets. Here, we consider “passage” as a token or a sequence of tokens that were assigned a label, for example, “triethylamine” annotated as *reagent_catalyst* and *other_compound* and “sodium hydrogen carbonate” annotated as *reagent_catalyst* and *other_compound* in ChEMU dataset. As more than one class is assigned to the same passage, we expect that they would be more ambiguous and therefore harder for the models to recognize. After the analyses of the training set, we notice that the ChEMU and WNUT corpora include passages that were assigned two or more labels for almost 10% of the examples. This happens for around only 1% of the annotations in the DEFT corpus. Hence, we would expect a better performance for the latter compared with the former. As it is not the case, it seems that the deep masked language models might actually be able to recognize those passages correctly using contextual information.

## 5 Discussion

We compared the effectiveness of individual masked language models and ensemble models based on the majority vote strategy for the NER task in multiple health and life science domains and languages. The ensemble model showed a robust performance across the assessed domains and languages, achieving an overall macro F1-score of 76.94% and improving the individual models by 6.0 percentage point (considering the BERT-based-cased, BERT-base-multilingual-cased, and Bio + Clinical BERT as reference for the individual models in the ChEMU, DEFT, and WNUT datasets, respectively) (*p* = 0.005). Out of the 38 entity classes assessed, 50% had an F1-score equal or higher than 85% for the ensemble model (compare to 34% for the individual BERT model).

The performance of the models on the French clinical corpus is lower than on the chemical and wet lab corpora. We believe this is likely due to the known issue of reduced French language resources compared to English, both in terms of the corpora to pretrain the masked language models and also to fine-tune for the clinical NER. As seen in entity distribution tables (Tables 1, 2, and 3), the training data for chemical and wet lab NER are larger, which results in better performance for the individual language models and consequently for the ensemble models. Additionally, the clinical dataset includes nested entities, which are known to be recognized more effectively using graph-based models (Yu et al., 2020). Nevertheless, it is for the clinical dataset that we notice the highest relative gain in performance for the ensemble model (9.5% of F1-score).

Our analysis shows that specialized language models achieve the best performance across the health and life science domains. Moreover, in terms of model architecture, BiLSTM-CRF-based models with contextualized language models for feature extraction achieve competitive results. These results are aligned with the current knowledge available in the literature (Fu et al., 2020b; Hahn and Oleynik, 2020). That said, existing methods for chemical, clinical, and wet lab NER focus mostly on a single domain and language. Here, we introduced a novel and generic NER methodology for diverse and complex corpora in multiple domains and languages. We believe that such an approach can be expanded to other domains and languages with similar effectiveness.

The detailed analysis of entity types shows that the models have often difficulties recognizing infrequent entities, such as *dose* (clinical corpus) and *Generic-Measure* (wet lab corpus), which is in-line with previous work (Fu et al., 2020a). However, we notice that for some entities, particularly in the wet lab corpora, the highest scores were provided by infrequent entities. Indeed, as shown by Fu et al. (2020a), a single holistic measure of F1-score cannot tell the details of the performance of different models. Diverse entity attributes, such as *length*, *frequency*, *sentence length*, and *out-of-vocabulary (OOV) density*, are important for further model analyses. Thus, we further examined three meta-features: entity frequency and length, and label consistency. There was a consistent performance gain brought by the ensemble as the entity length increased. As deep masked language models work at the sub-word level, the longer the entity size, the more correct classifications are needed by the individual model to provide an exact match NER. By combining the different models, the ensemble seems to be able to leverage the correct classifications among the models. Moreover, despite a relatively frequent multi-labeling for passages (2 or more) in the chemical and wet lab corpora compared to the clinical corpus, their performance was significantly higher than the latter. This result suggests that, as expected, the deep masked language models were able to distinguish the homographs by their context.

The main limitation of our results comes from the heterogeneity of both corpora and models used. We used different baseline models across domains, partly due to the nature of the datasets (different languages). Additionally, the distribution of entities differs significantly across the datasets. All of this hinders the comparison of the results. Nevertheless, we believe the overall methodology gives a strong indication of the robustness of the ensemble of deep language models for NER in multi-domain and -lingual corpora.

## 6 Conclusion

In this work, we propose a generic and robust approach for named entity recognition in the health and life science domain based on deep masked language models combined in a majority voting strategy. We compared the performance of individual BERT models and their siblings against the proposed ensemble models for three types of corpora—chemical, clinical, and wet lab—available in English and French languages. We show a significant performance improvement of 6.0 percentage point (*p* = 0.005) using the ensemble models compared to a strong baseline based on individual BERT models, with the ensemble models having 50% of entities assessed with an F1-score of 85% or more. We further performed a detailed analysis of the performance of the models based on a set of entity properties. We found that ensemble models can be more beneficial for longer entities.

## Data Availability

Publicly available datasets were analyzed in this study. The data used for chemical NER can be found at: http://chemu2020.eng.unimelb.edu.au/, https://deft.limsi.fr/2020/, http://noisy-text.github.io/2020/wlp-task.html.

## Footnotes

1
https://bitbucket.org/nicta_biomed/brateval/
